# Dengue virus and the 2024 Paris Olympics

**DOI:** 10.3389/fpubh.2024.1452758

**Published:** 2024-08-16

**Authors:** Milad Zandi, Ismaeil Alizadeh, Fatemeh Sadat Mousavi, Maryam Faraji

**Affiliations:** ^1^Department of Microbiology, Faculty of Medicine, Guilan University of Medical Sciences, Rasht, Iran; ^2^Department of Vector Biology and Control of Diseases, School of Public Health, Tehran University of Medical Sciences, Tehran, Iran; ^3^Department of Microbiology and Immunology, Faculty of Veterinary Medicine, University of Tehran, Tehran, Iran; ^4^Environmental Health Engineering Research Center, Kerman University of Medical Sciences, Kerman, Iran

**Keywords:** dengue, Paris 2024 Olympics, mosquito-borne diseases, health measures, arbovirus

## Abstract

The 2024 Paris Olympics and Paralympics face concerns over dengue virus transmission, despite Paris’s lower mosquito activity. Preventive measures include eliminating breeding sites, insecticide spraying, and public awareness. Health systems will monitor and respond to cases. Large gatherings like the Olympics can amplify disease spread, as seen with Zika in Rio 2016. Recent reports confirm dengue presence in Europe, highlighting global risks. While Paris’s overall dengue risk is low, even a few cases could impact global health. Collaboration among health authorities, researchers, and event organizers is crucial to ensure participant and public safety during the games.

## Introduction

As the world eagerly anticipates the start of the 2024 Olympic Games in Paris, scheduled from July 26 to August 11, and the Paralympic Games from August 28 to September 8, there is a growing concern about the safety and health of those attending the games, particularly regarding the dengue virus. Dengue, a mosquito-borne illness transmitted primarily by the *Aedes aegypti* mosquito, has seen sporadic outbreaks in the southern regions of France due to climate change and increased international travel (1). Symptoms of dengue, caused by the dengue virus (DENV), can range from mild fever and joint pain to severe and potentially fatal hemorrhagic fever if not treated properly. These symptoms can also include life-threatening complications such as dengue shock syndrome (DSS), characterized by a sudden drop in blood pressure (2). Other common symptoms of dengue include severe headache, pain behind the eyes, nausea, vomiting, swollen glands, rash, and severe muscle and bone pain, often called “breakbone fever.” In severe cases, patients may experience bleeding gums, blood in vomit or stool, and difficulty breathing (3, 4). However, Paris, with its temperate climate, is less prone to mosquito proliferation compared to the southern parts of the country. The games are set to take place in late summer, a period when mosquito activity is generally on the decline. This significantly reduces the risk of dengue transmission during the Olympics. The French health authorities and Olympic organizers have implemented rigorous preventive measures to ensure the safety of all participants and spectators. These measures include intensive efforts to eliminate mosquito breeding sites, regular spraying of insecticides in and around Olympic venues, and public awareness campaigns on how to prevent mosquito bites through the use of insect repellents and wearing protective clothing. Additionally, health monitoring systems will be in place to promptly identify and respond to any reported cases of dengue, with medical facilities in Paris well-equipped to handle such infectious diseases.

However, the potential risks posed by large international gatherings, like the Olympic Games, underscore the presence of arboviral diseases globally transmitted by arthropods, including dengue, Zika, chikungunya, yellow fever, and West Nile infection (5). During the Rio 2016 Olympics in Brazil, the re-emergence of the Zika virus, despite its low incidence, posed significant challenges for event authorities, emphasizing the need for robust preparedness and response strategies in the face of emerging infectious diseases during large-scale international gatherings (6). The upcoming Paris 2024 Olympics are anticipated to attract millions of international visitors, highlighting the need for vigilance and proactive measures to safeguard the health of participants and attendees. Collaboration between health authorities, researchers, and event organizers is crucial in addressing and mitigating any potential public health challenges associated with dengue during the games. The presence of dengue vectors, including *Aedes aegypti* and *Aedes albopictus*, in Europe has raised considerable concern. Reports from 2022 and 2023 indicate the presence of dengue fever in Europe, with autochthonous cases documented in countries such as France, Spain, and Italy. France has reported cases of dengue since 2010, with the vector *Aedes albopictus* spreading northward from the south since it was first detected in 2004. In 2022 and 2023, France reported 65 and 43 cases of dengue, respectively, including cases near Paris (7).

The risks associated with the Paris Olympics are multifaceted. The games attract visitors from every country, creating a unique potential for disease spread. If visitors become infected with dengue, even asymptomatically, and return to their home countries, particularly tropical regions with suitable conditions for *Aedes aegypti* mosquitoes, this could establish local viral transmission and lead to new outbreaks. Regions in Africa and Asia, which already face challenges with mosquito-borne diseases and have weaker health systems, could be particularly vulnerable. Although the estimated 15,000,000 visitors to the Olympics may seem minor compared to global travel numbers, history shows that even a small number of infected travelers can have significant impacts, as seen with Brazil’s Zika outbreak (8). While the overall risk of dengue transmission at the Paris Olympics is low, the potential consequences of even a few cases spreading globally underscore the need for vigilant preventive efforts and comprehensive health advisories for all attendees. The collaboration between health authorities, researchers, and event organizers remains crucial in ensuring that preventive measures are implemented effectively, and the health of both participants and local populations is safeguarded. The limitations of this article include a narrow temporal and geographical focus and reliance on recent, limited data, which may not fully capture long-term trends or the broader regional impact. Additionally, assumptions about the effectiveness of preventive measures and the primary focus on dengue may overlook other concurrent disease risks.

## Conclusion

The 2024 Olympic and Paralympic Games in Paris bring concerns about dengue virus transmission. Despite Paris’s low risk due to its temperate climate and the timing of the games, preventive measures are in place to mitigate potential risks. The influx of international visitors poses a multifaceted disease spread risk, underscoring the need for vigilance and collaboration between health authorities, researchers, and event organizers to safeguard public health. The overall risk is low, but the potential global impact of a few cases necessitates effective preventive efforts and health advisories.

## Data Availability

The original contributions presented in the study are included in the article/supplementary material, further inquiries can be directed to the corresponding author.
